# Cardiac rhabdomyomas as prenatal diagnostic markers of tuberous sclerosis complex^☆^

**DOI:** 10.1016/j.abd.2021.10.019

**Published:** 2023-07-20

**Authors:** Virginia Ruth Lopez Gamboa, Mariel Giovo, Victor Francucci

**Affiliations:** a Departmente of Dermatology, Collegiate Sanatorium, CABA, Argentina; b Department of Dermatology, Holy Trinity Children's Hospital, Cordoba, Argentina; c Department of Dermatology, Neonatal Maternal Hospital, “Minister Dr. Ramon Carrillo”, Cordoba, Argentina; d Private practice, Buenos Aires, Argentina

Dear Editor,

Tuberous Sclerosis Complex (TSC) is a rare genetic neurocutaneous syndrome, with a frequency of 1/6,000‒10,000 live births, characterized by hamartomas and multiple skin manifestations.^1^ Adequate diagnosis is challenging, therefore the TSC Alliance^2^ convened on criteria, which include cardiac rhabdomyomas, a type of hamartomas, as a main diagnostic feature.^3^ These tumors are diagnosed via ultrasound during the second and third trimester, correlating with TSC in 70%–90% of the cases.^3^, ^4^

The authors present three male patients with a prenatal diagnosis of cardiac rhabdomyomas and postnatal confirmation of TSC. Dermatologic examination of all patients revealed multiple hypopigmented macules in the trunk and scalp (Fig. 1), more evident under Wood’s lamp (Fig. 1). Patient A had no family history of TSC and presented fetal arrhythmia caused by multiple cardiac tumors located in the left ventricle, which regressed during the first year of life. Patient B presented a fetal asymptomatic solitary rhabdomyoma, which also regressed during the first year. In this case, the authors noticed his mother had multiple hamartomas of the face (Fig. 2) so after further examination, she was also diagnosed with TSC. Patient C had three cardiac rhabdomyomas diagnosed in the third trimester causing cardiac flow obstruction. Three months after birth, he was admitted due to seizures, which led to the confirmation of tuberous tumors in the brain and retinal hamartomas. Despite medical efforts, he had a fatal outcome. The diagnosis of TSC in all patients was based on two major clinical criteria:^3^ hypomelanotic macules (≥3, at least 5 mm in diameter) and cardiac rhabdomyomas.

**Figure 1:**
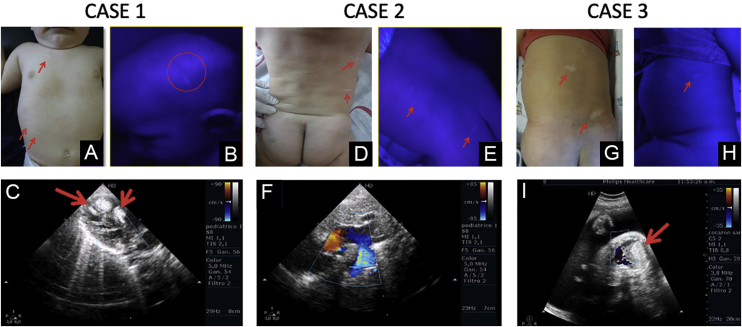
(A‒I) Clinical images at physical examination, under Wood´s lamp and sonographic imaging of cardiac rhabdomyomas of each case

**Figure 2:**
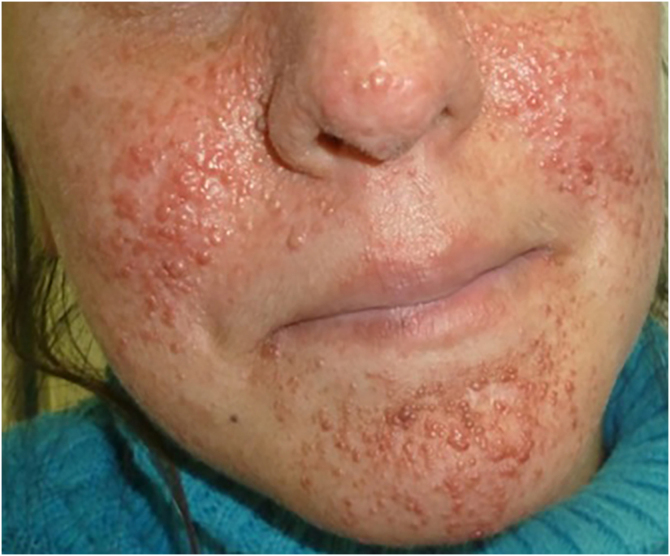
Case B´s mother shows multiple hamartomas of the face

Cardiac rhabdomyomas are the most frequent childhood primary heart tumors in the general population.^3^ Despite their benign nature, they may cause complications such as arrhythmias, outflow obstruction, pericardial effusion, cardiac compression, and fetal hydrops. TSC should be suspected when multiple, clearly demarcated, hyperechoic ovoid tumors are found. The most frequent location is the interventricular septum. If possible, searching for hamartomas in other locations with fetal MRI is suggested.^1^, ^5^ Also, genetic testing assessment of three generations, along with a thorough dermatologic exam of relatives is indicated. When performed, a histological description of the tumors presents myocytes containing high quantities of glycogen, known as spider cells.^3^

Fetal prognosis is dependent on effective intrauterine monitoring and an adequate birth plan. Up to 85% of the cases do not need medical or surgical treatment since most have partial or complete regression from birth to adolescence.^5^ While skin findings might not be evident at birth, periodic dermatology consults are required in the search for angiofibromas, ungueal fibromas, hypomelanotic macules and/or Shagreen patch.^3^ These cases exemplify the importance of identifying rhabdomyomas, to help prepare for postnatal care, counsel parents and through adequate family history, identify affected relatives^1^, ^3^ of this rare neurocutaneous syndrome.

## Financial support

None declared.

## Authors' contributions

Virginia Ruth Lopez Gamboa: Approval of the final version of the manuscript; critical literature review; data collection, analysis, and interpretation; effective participation in research orientation; intellectual participation in propaedeutic and/or therapeutic management of studied cases; manuscript critical review; preparation and writing of the manuscript; study conception and planning.

Mariel Giovo: Approval of the final version of the manuscript; critical literature review; data collection, analysis, and interpretation; effective participation in research orientation; intellectual participation in propaedeutic and/or therapeutic management of studied cases; manuscript critical review; study conception and planning.

Victor Francucci: Approval of the final version of the manuscript; data collection, analysis, and interpretation; effective participation in research orientation; manuscript critical review.

## Conflicts of interest

None declared.
